# Lesion classification and diabetic retinopathy grading by integrating softmax and pooling operators into vision transformer

**DOI:** 10.3389/fpubh.2024.1442114

**Published:** 2025-01-06

**Authors:** Chong Liu, Weiguang Wang, Jian Lian, Wanzhen Jiao

**Affiliations:** ^1^School of Intelligence Engineering, Shandong Management University, Jinan, China; ^2^Department of Ophthalmology, Shandong Provincial Hospital Affiliated to Shandong First Medical University, Jinan, China

**Keywords:** medical image analysis, image classification, deep learning, Bi-LSTM, transformer

## Abstract

**Introduction:**

Diabetic retinopathy grading plays a vital role in the diagnosis and treatment of patients. In practice, this task mainly relies on manual inspection using human visual system. However, the human visual system-based screening process is labor-intensive, time-consuming, and error-prone. Therefore, plenty of automated screening technique have been developed to address this task.

**Methods:**

Among these techniques, the deep learning models have demonstrated promising outcomes in various types of machine vision tasks. However, most of the medical image analysis-oriented deep learning approaches are built upon the convolutional operations, which might neglect the global dependencies between long-range pixels in the medical images. Therefore, the vision transformer models, which can unveil the associations between global pixels, have been gradually employed in medical image analysis. However, the quadratic computation complexity of attention mechanism has hindered the deployment of vision transformer in clinical practices. Bearing the analysis above in mind, this study introduces an integrated self-attention mechanism with both softmax and linear modules to guarantee efficiency and expressiveness, simultaneously. To be specific, a portion of query and key tokens, which are much less than the original query and key tokens, are adopted in the attention module by adding a set of proxy tokens. Note that the proxy tokens can fully utilize both the advantages of softmax and linear attention.

**Results:**

To evaluate the performance of the presented approach, the comparison experiments between state-of-the-art algorithms and the proposed approach are conducted. Experimental results demonstrate that the proposed approach achieves superior outcome over the state-of-the-art algorithms on the publicly available datasets.

**Discussion:**

Accordingly, the proposed approach can be taken as a potentially valuable instrument in clinical practices.

## 1 Introduction

As a chronic disease, diabetes occurs either when the body cannot produce enough insulin or cannot effectively use the insulin (1). Diabetes has an influence on millions of people around the world in every year. In the past decades, there have been more than 450 million people considered to suffer from either Type 1 or Type 2 diabetes (2). Diabetes can cause damage to multiple human organs, including eyes, heart, kidneys, and nerves. Diabetic retinopathy (DR) is extensively considered as long-term complication caused by diabetes. DR, which is accepted as a significant causes of blindness globally, can adversely affect the blood vessels in the retina and even result in total vision loss (3). About 30 percents of the patients with diabetes will develop DR, which usually remains undiscovered until DR has reached a certain stage when the effective treatment is unable to receive.

The development of DR can be roughly categorized into different stages, which are determined by a variety of structural lesions appearing in fundus images. Typically, the lesions consists of the followings: hard exudate, soft exudate, hemorrhage, microaneurysm, and neovascularization. These lesions usually manifest as minute objects in retinal images and make a huge contribution to DR grading in clinical practices. Accordingly, accurate detection of these lesions and accompanying DR grading pose a great challenge to the medical diagnosis (4, 5). Meanwhile, tiny DR lesions at early stage can also be difficult to recognize since they are hard to to distinguish from blood vessels. Currently, the treatments for DR rely on accurate DR grading to slow down the development of vision impairment. However, manual identification of DR lesions in retinal images is time-consuming, error-prone, and labor-tedious. In addition, the underdeveloped areas usually lack of ophthalmologists and advanced equipment to implement DR grading manually. Therefore, automatic DR grading in retinal images has attracted considerable attention in the field of both machine vision and ophthalmology. DR lesion detection includes the recognition of various types of lesions, and DR grading aims at evaluating the severity of DR in a retinal image. Both tasks play a vital role for early diagnosis of DR.

In recent years, the deep learning models have shown their promising outcomes in a great deal of areas, including machine vision and natural language processing. In addition, numerous endeavors have been made to DR lesion detection and DR grading. After the early work of Google in 2016 for classification of DR in fundus images, Hunt et al. (6) presented a low-shot, self-supervised deep learning method for classification of retinal fundus images. The low-shot mechanism of learning in this work resolved the problem of insufficient image samples. To implement the detection of DR at its early stage, the study of (7) proposed an investigation of the applications of deep learning models for retinal image classification. In general, the deep learning architectures, including the conventional convolutional neural network (CNN) and other deep CNNs, were incorporated in this survey. In the work of Tak et al. (8), a deep CNN model was trained to classify diseased retinal images. Accordingly, 420 wide-field retinal images were included in the training process for discriminating the exudate and non-exudate cases. Umamageswari et al. (9) proposed an approach to identify exudates and veins with retinal images for diagnosing diabetics. Specifically, a CNN was proposed for retinal image recognition. Recently, to segment and classify the retinal images in a unified way, Kumari et al. (10) proposed an efficient CNN model. The input images for the proposed model were pre-processed using the green channel images, histogram-based algorithms, and noise elimination techniques. The features were extracted from the segmented images using the watershed algorithm as well as principal component analysis (PCA) technique. Meanwhile, the publicly available datasets used in this study are DRIVE (11), STARE (12), and CHASE DB1 (13). Moreover, Ilesanmi et al. (14) systematically reviewed the applications of CNN in both segmentation and classification of fundus images.

Different from the CNN-based models, a great deal of vision transformer-based models have been proposed to deal with retinal image classification tasks. Note that the vision transformer models can eliminate the disadvantages of convolutional modules, such as local receptive fields. For instance, Wang et al. (15) presented a vision transformer model called retinal ViT, which incorporates the self-attention mechanism into the field of medical image analysis and has outperformed the state-of-the-art algorithms in terms of various evaluation metrics. Yang et al. (16) attempted to classify referable DR based on large-size retinal images using a vision transformer. A vision transformer with masked autoencoders (MAE) was applied to improve the classification performance. Karn and Abdulla (17) presented a model called the dual-scale twin vision transformer for retinal disease classification using OCT images. This model combines the advantages of dual-scale representation learning and the twin transformer architecture to improve disease classification accuracy.

Bearing the above-mentioned analysis in mind, this study proposes a novel vision transformer model to implement lesion detection and DR grading in an end-to-end fashion. Note that the attention mechanism in the proposed model can reveal the global associations between long-range pixels in the images. On the other hand, the proposed attention module can significantly reduce its consumption of computing resources during the calculations of global associations between distant pixels. In general, a novel attention mechanism is introduced into the conventional vision transformer model (18). It is notable that the proposed attention module is inspired by the work of Han et al. (19), which provides the agent attention module. Then, the proposed attention module integrates both the softmax and pooling operations. In addition, the presented approach exploits the idea of agent attention (19) while adding a different type of agent token. To be specific, the proposed vision transformer model first generates the query tokens, key tokens, and value tokens. Then, the agent tokens are down-sampled from the query tokens to represent the global information of the images. Meanwhile, in addition to the agent tokens for query tokens, the agent tokens for key tokens are also leveraged to capture the local features from the images. In the following process, both the query agent tokens and key agent tokens are integrated and broadcasted to the query tokens, which can further enhance the expressiveness of the proposed vision transformer. Note that the positional token can be incorporated into the proposed architecture to provide position information for the features. To evaluate the proposed approach, we conducted comparison experiments between state-of-the-art methods and our method on the publicly available datasets for both lesion classification and DR grading. Experimental results of the proposed method demonstrate the superiority of this work over the state-of-the-art in terms of a set of evaluation metrics. The main contributions of this study can be summarized as follows:

In general, the main contributions of this study can be summarized as:

- A novel vision transformer-based pipeline for retinal image classification is proposed.
- Due to the characteristics of retinal images, an integration of softmax and linear attention was presented.
- Experimental results demonstrate the value of the proposed model in clinical practice.

The rest of this article is summarized as follows: The specifics of the proposed pipeline are outlined in Section 2. Section 3 outlines the experimental details employed to evaluate the efficacy of the suggested technique. In addition, Section 3 also provides the discussion of this study. Finally, the study's conclusion is presented in Section 4.

## 2 Methodology

**Figure 1:**
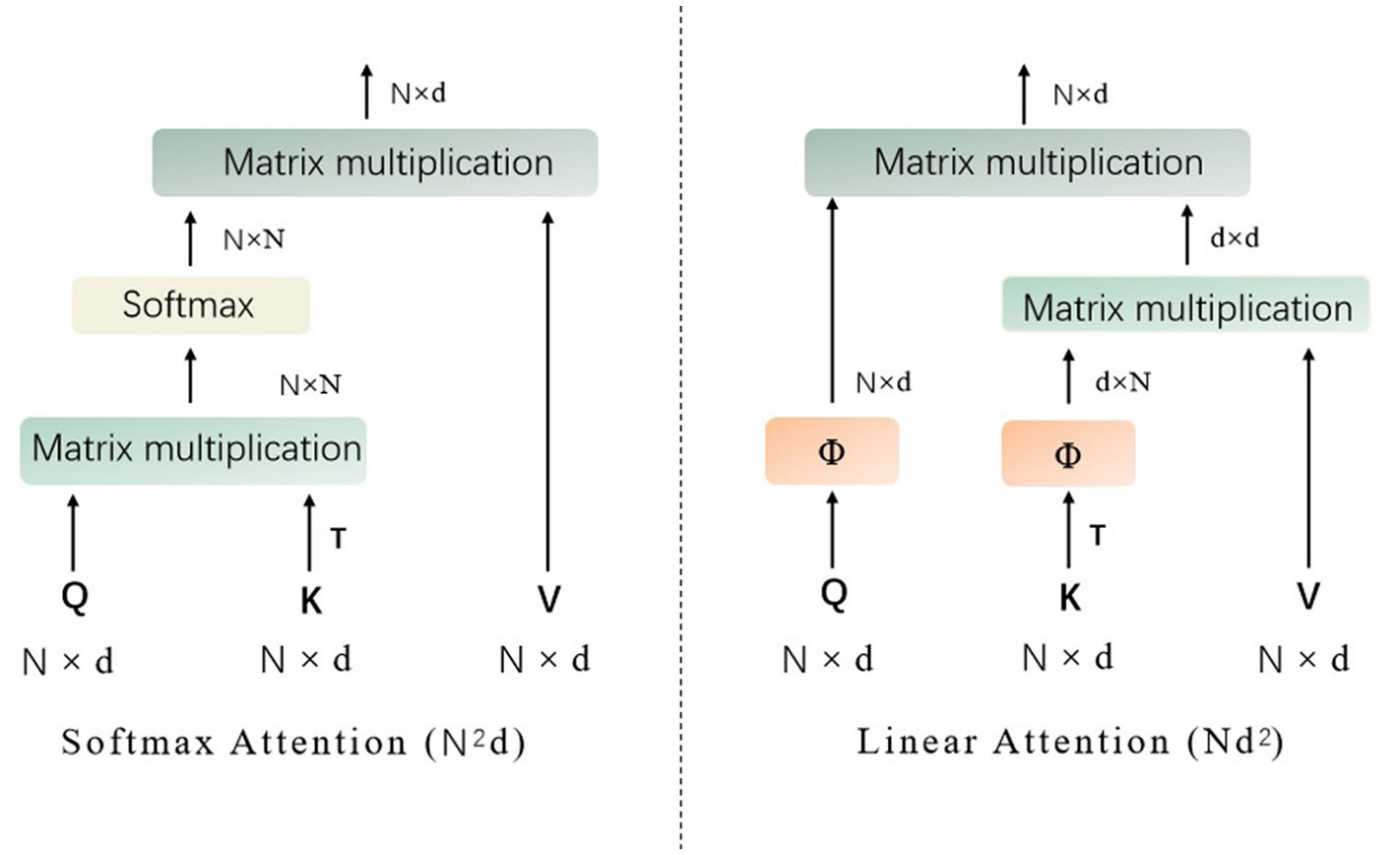
The structures of Softmax attention and linear attention (Q represents the query tokens, K denotes the key tokens, V are the value tokens, T denotes matrix transpose operation, and Softmax denotes the the softmax function).

In this section, we mainly describe the details about the proposed approach based on vision transformer. To be specific, to address lesion classification and DR grading in retinal images, we introduce a novel attention mechanism, inspired by the work of agent attention (19), into the vision transformer (18) architecture. As depicted in the following content, the original agent attention (19) is an attention paradigm that integrates the strengths of both Softmax and linear attention mechanisms, as shown in Figure 1.

**Figure 2:**
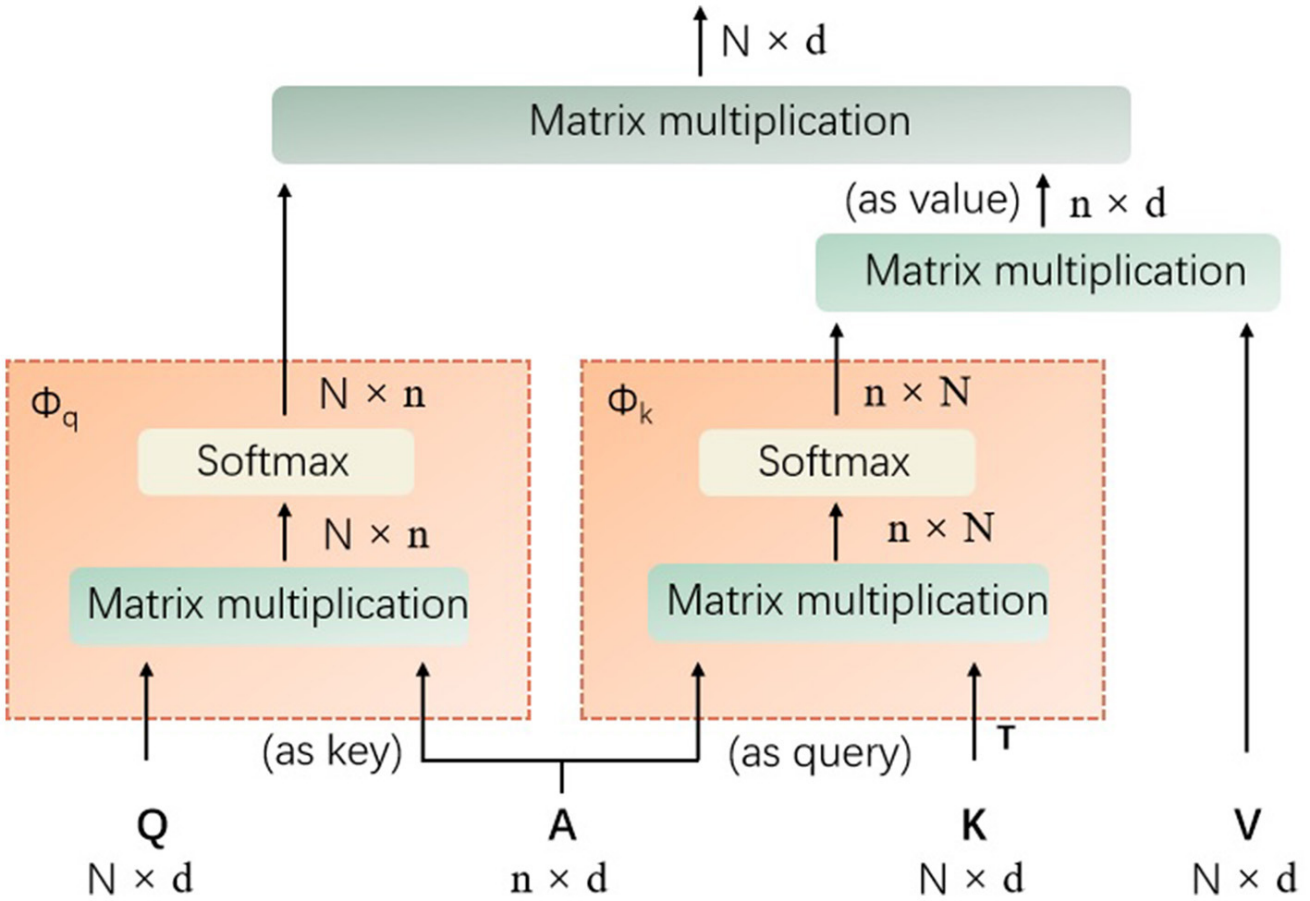
The details of the introduced agent attention (Q represents the query tokens, A are the agent tokens, K denotes the key tokens, V are the value tokens, T denotes matrix transpose operation, and softmax denotes the the softmax function).

It is designed to balance computational efficiency with representational power, particularly useful for vision tasks involving transformers. Mathematically, the agent attention can be described as a two-step process involving agent aggregation and agent broadcasting, denoted as a quadruple (Q, A, K, V), where Q represents the query tokens, A are the agent tokens, K are the key tokens, and V are the value tokens, as shown in Figure 2.

**Agent aggregation:** In its first step, the agent tokens A aggregate information from the key-value pairs (K, V). This is achieved through a self-attention mechanism with A acting as queries, and (K, V) as key-value pairs. The output of this step is denoted as *V*_*A*_, which is calculated as Equation 1:


(1) $$\begin{array}{l} V_{A}=softmax\left(\frac{AK^{T}}{\sqrt{d}}\right)V \end{array}$$

As shown in Equation 1, the softmax denotes the softmax function, d is the dimensionality of the embeddings, and the operation aggregates the values associated with each agent token based on the softmax attention over the keys.

**Agent broadcasting:** The second step involves broadcasting the aggregated information back to the original query tokens Q. This is done by treating Q as queries and *V*_*A*_ as key-value pairs. The mathematical expression of the final output O is provided in Equation 2:


(2) $$\begin{array}{l} O=softmax\left(\frac{QA^{T}}{\sqrt{d}}\right)V_{A} \end{array}$$

In this procedure, each query token receives a weighted sum of the aggregated agent features, with weights determined by the softmax attention between Q and the agent tokens. The power of agent attention lies in its ability to leverage a smaller number of agent tokens compared to the number of query tokens, which results in reduced computational complexity while maintaining the global context modeling capability.

To implement agent attention, the projection matrices need to be defined to transform the input tokens into query, key, and value representations, respectively. The agent tokens A are then obtained through a pooling operation on the query tokens Q. The two-step attention process is then applied as described above, with the softmax function computing the attention weights and the resulting outputs being passed through a feed-forward network to produce the final output.

### 2.1 Proxy attention: the improved agent attention

**Figure 3:**
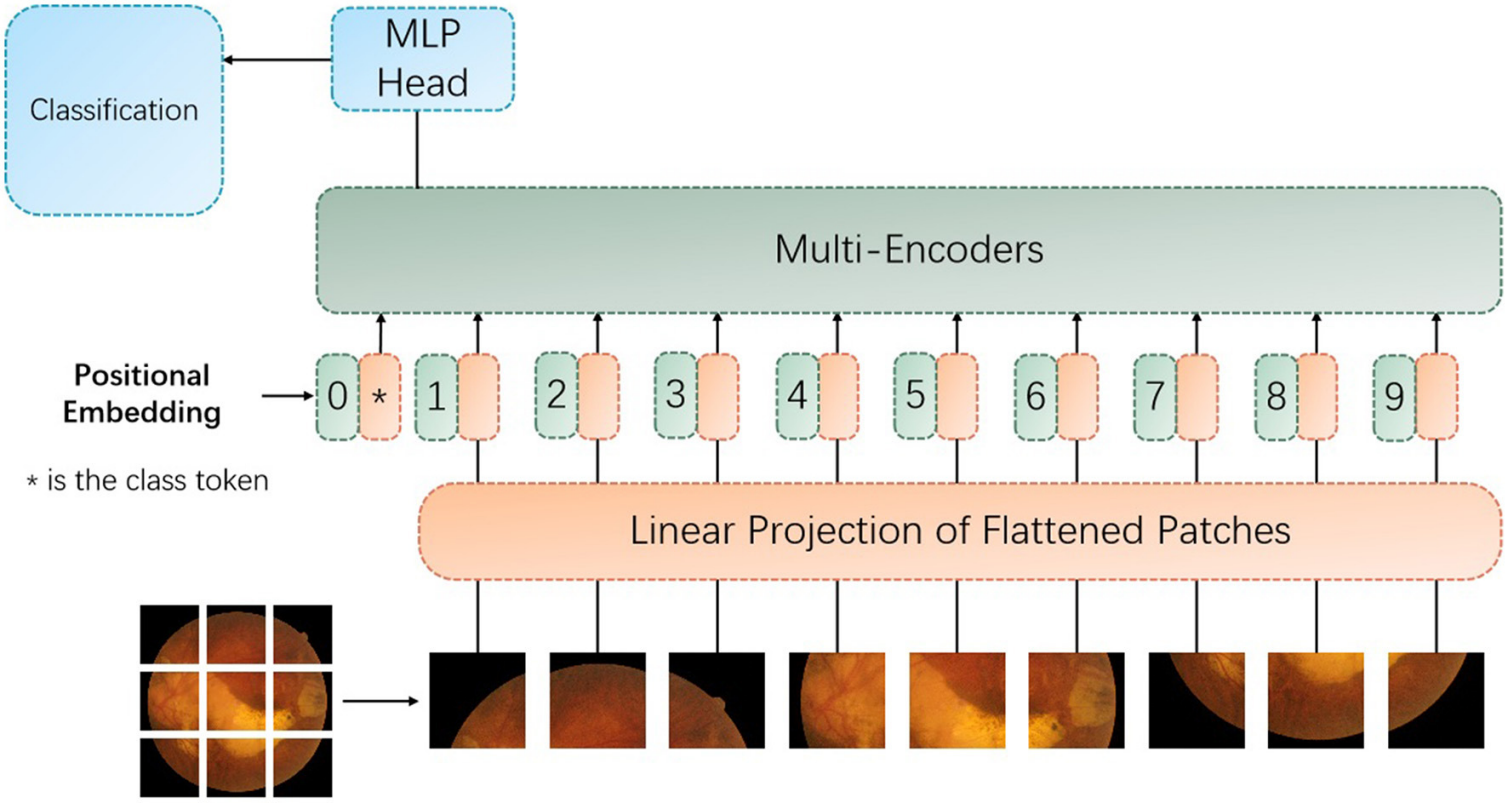
The architecture of the proposed vision transformer with the improved encoder with the proxy attention. MLP denotes multilayer perceptron.

To further improve the performance of the proposed approach, we introduce proxy token, which is based on the key token used in the original agent attention (19) module. This would require subtle adjustments and innovations to the original agent attention framework. The purpose of doing this is to further enhance the model's ability to capture local features and to enhance the performance of the proposed vision transformer (as shown in Figure 3) model by adding different types of agent tokens.

The proxy attention mechanism is designed to leverage the strengths of both global and local attention. To be specific, the first set of proxy tokens, derived from the query tokens, is effective for capturing the global context of the image. However, for tasks like retinal image analysis, where fine-grained details are crucial, the local features are just as important. Retinal images contain a wealth of local features that are indicative of specific pathologies. For instance, microaneurysms in diabetic retinopathy appear as small, localized bright spots, and exudates can manifest as small, yellowish deposits. Traditional global attention mechanisms might overlook these subtle local features due to their focus on the overall image context. The key tokens in the transformer architecture can be used to capture the presence and relationships of features across the entire image. By generating a second set of agent tokens directly from the key tokens, the proposed model can afford a more nuanced exploration of these features. To note that the second set of agent tokens acts as a spotlight, directing the model's focus toward the intricate local patterns and textures that are indicative of specific retinal pathologies.

**Figure 4:**
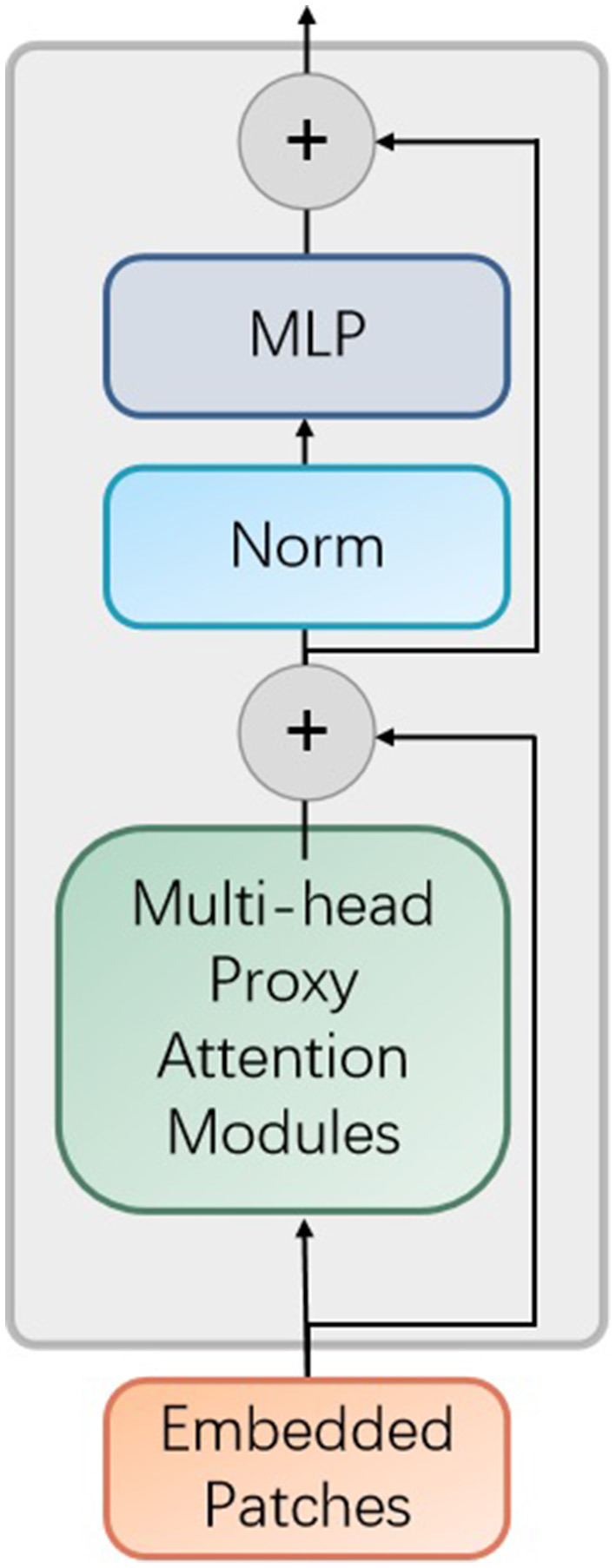
The improved encoder module in the proposed vision transformer model. MLP denotes multilayer perceptron and Norm represents normalization operator.

As demonstrated in Figure 3, the multi-encoders are composed of the proposed encoder, as provided in Figure 4.

**Figure 5:**
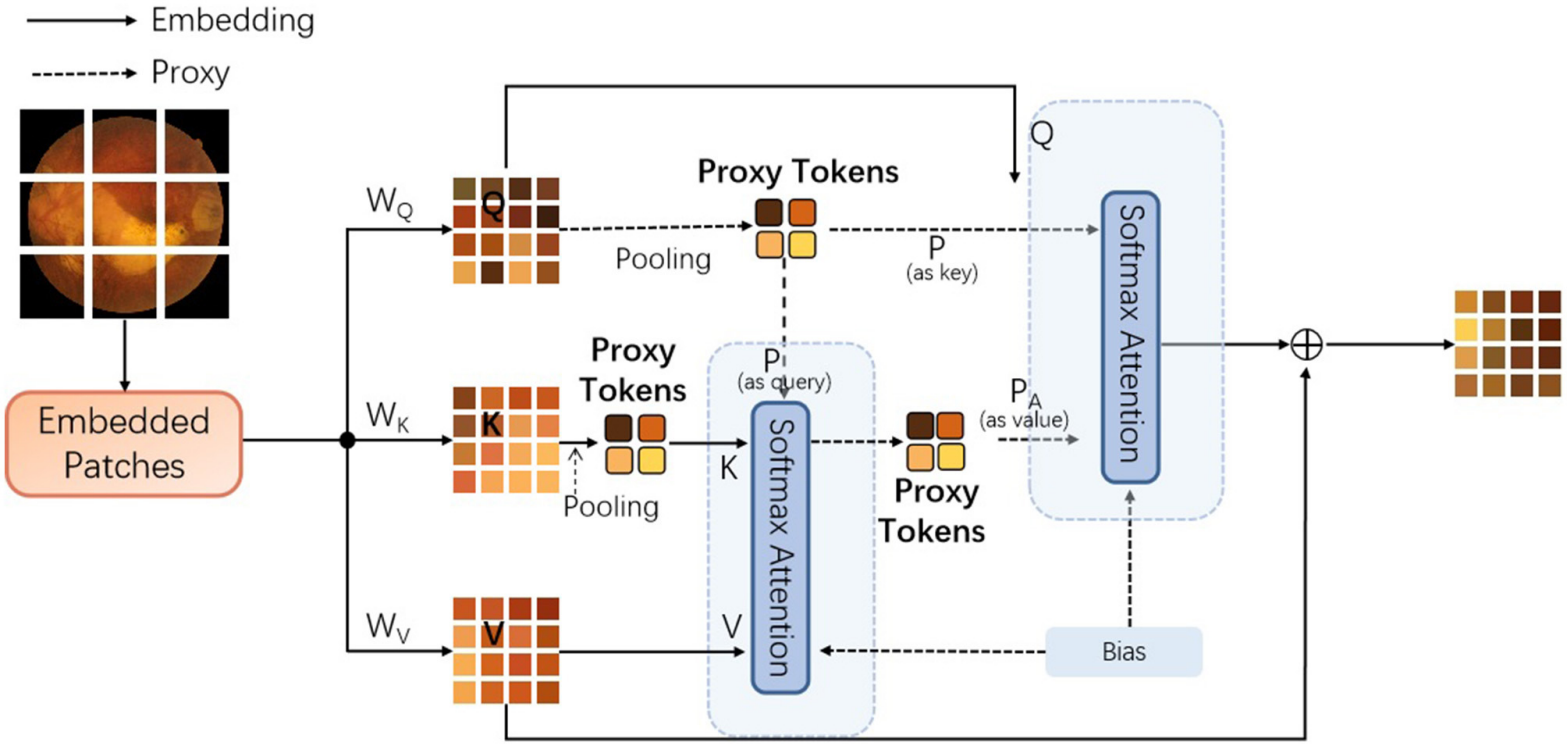
The details of the improved agent attention module, dubbed proxy attention [Q represents the query tokens, K denotes the key tokens, and V are the value tokens. *W*_*Q*_, *W*_*K*_, and *W*_*V*_ denote the weighting matrices for queries, keys, and values. In addition, the Bias denotes the bias component inspired by the work of (20). To note that the pooling operations are both max-pooling].

In addition, each encoder contains the proposed proxy attention module, as shown in Figure 5.

To incorporate the Key-based agent tokens, the following steps need to be incorporated: First, retain the original agent tokens generated from the query tokens. These agent tokens are responsible for aggregating global information; Secondly, a new mechanism can be designed to generate a second set of agent tokens from key tokens. This set of agent tokens can focus on capturing local or specific types of feature information; Then, during the attention computation process, these two sets of agent tokens can be combined, in order to utilize both global and local information, simultaneously. In this process, the model we propose initially extracts features from the input image through a feature extraction layer, and then generates query tokens (Q), key tokens (K), and value tokens (V). Subsequently, we aggregate Q to generate the original proxy tokens (A1), which represent the global information of the image. In parallel, we also generate proxy tokens based on Keys (A2) from K, which may focus on capturing local features or specific types of information within the image. In addition, the algorithm flowchart of this process is provided in Algorithm 1.

**Algorithm 1:** Proxy attention mechanism.

1: procedure ProxyAttention(*Q, K, V*)
2: *A*_1_ ← Downsample(*Q*) ⊳ Generate proxy tokens for global information
3: *A*_2_ ← Downsample(*K*) ⊳ Generate proxy tokens for local features
4: *V*_*A*_ ← SelfAttention(*A*_1_, *K, V*) ⊳ Agent aggregation
5: *O* ← SelfAttention(*Q, A*_1_, *V*_*A*_) ⊳ Agent broadcasting
6: *O* ← *O* + SelfAttention(*Q, A*_2_, *V*) ⊳ Incorporating local features
7: return *O*
8: end **procedure**

To note that the traditional attention mechanisms, including those used in vision transformers, have a quadratic complexity with respect to the sequence length due to the need to compute pairwise interactions between all tokens. The proposed attention mechanism mitigates this by reducing the number of tokens through pooling operations before computing attention, thus transforming the quadratic complexity into a more manageable linear complexity. It should be noted that this improvement may increase the complexity and computational burden of the model because it introduces additional agent tokens and corresponding computational processes. Therefore, in practical applications, it is necessary to weigh the relationship between the performance gains brought about by this improvement and the additional costs, and to decide whether to adopt this hybrid agent token approach based on specific tasks and resource constraints.

## 3 Experiments

### 3.1 Dataset

In this section, we sequentially provide the experimental settings, ablation studies, and the experimental results using the proposed approach and the competing algorithms. The proposed vision transformer-based model was deliberately crafted using renowned public datasets for Diabetic Retinopathy (DR) grading, specifically the APTOS2019 database (21) and the Messidor dataset (22). The APTOS2019 database encompasses a collection of 3,662 fundus images, each annotated with one of the five grades of DR, as detailed in Table 1. In alignment with prevailing benchmarks (23–27), we employed a rigorous 10-fold cross-validation framework to assess the performance of our model on the APTOS2019 dataset.

**Table 1:** The detailed distribution of APTOS2019 and Messidor datasets.

**Class**	**No. of images in APTOS2019**	**No. of images in Messidor**
DR 0	1,805	546
DR 1	370	153
DR 2	999	247
DR 3	193	254
DR 4	295	-
Total	3,662	1,200

Conversely, the Messidor dataset comprises 1,200 fundus images, complete with DR grading and diabetic macular edema (DME) annotations, as outlined in Table 1. For a fair comparison with existing literature (25, 28), we focused on images classified as DR grades 0 and 1 from the Messidor dataset, utilizing a 10-fold cross-validation approach for this binary classification task. Moreover, we harnessed the complete Messidor dataset to refine our model for the DR grading task, thereby enhancing its clinical applicability and robustness.

### 3.2 Implementation details

For the pre-training of our proposed vision transformer model, we utilized the expansive ImageNet ISLVRC2012^1^ dataset, which serves as a rich repository of natural images. This dataset comprises over 120 million images spanning 1,000 distinct classes. To align with the requirements of the vision transformer architecture, we resized the images from the ImageNet ISLVRC2012 dataset to a uniform resolution of 256 × 256 pixels for the duration of the pre-training phase. Our model's initialization diverges from that of the original vision transformer as presented in (18), in that we did not employ pre-trained weight parameters. Instead, we chose to initialize our model's weight parameters based on a specific iteration of the ImageNet dataset (29), eschewing the traditional random initialization strategy.

The pre-training phase of our model is structured as a multi-classification task, with the objective function defined by the binary cross-entropy loss. This loss function is integral to the training process and is depicted in Equation 3 for clarity and reference. This approach ensures that our model is finely tuned to the characteristics and distribution of the natural image data present in the ImageNet ISLVRC2012 dataset, providing a robust foundation for subsequent fine-tuning and task-specific training.


(3) $$\begin{array}{c} Loss\left(y,y^{\prime }\right)=\overset{C}{\underset{i=1}{\sum }}y_{i}log\left(y_{i}^{\prime }\right), \end{array}$$

where *y* and *y*′ denote the ground-truth label and prediction of the label, respectively.

The hyper-parameters employed in our proposed vision transformer model are meticulously detailed in Table 2. We utilize both grid search and random search techniques to explore a comprehensive hyperparameter space. This included the learning rate, batch size, number of layers, and epochs. To address the varying sizes of image patches, we interpolate the corresponding position embeddings for each patch. This interpolation technique ensures that the model can effectively handle a diverse set of image patches by providing appropriate positional context, which is essential for the transformer's attention mechanism to function optimally. In the context of retinal image classification, our model is trained using the cross-entropy loss function, which serves as the supervisory signal for both the feature extractor and the classification module, as depicted in Equation 3. This loss function is instrumental in guiding the model to learn discriminative features that are critical for accurate classification of retinal conditions.

**Table 2:** The leveraged hyper-parameters values of the proposed approach.

**Parameter**	**Setting**
Batch size	8
Classes	5 (DR grading) or 2 (Binary)
Image resolution	256 × 256
Optimizer	Adam
Learning rate	1e-5
Patch size	16
Depth	12
Epochs	500

During the training phase, a comprehensive suite of data augmentation techniques is implemented to enhance the robustness and generalizability of the model. Data augmentation techniques are applied more aggressively to the minority class to artificially increase its representation in the training set. This includes transformations such as rotations, flips, and zooms, which help the model generalize better to new, unseen data. The development and training of the model are facilitated by the PyTorch framework (30), which is renowned for its flexibility and efficiency in deep learning applications. Our training infrastructure is bolstered by 4 NVIDIA Tesla V100 GPUs, which provide the computational prowess necessary for handling the complex operations involved in neural network training. On average, the model processes each image in 420 milliseconds, ensuring that the training pipeline is conducted in a timely and efficient manner.

In our experimental evaluation, we adopt a comprehensive set of metrics to thoroughly assess the performance of the model. These metrics include the Area Under the Curve (AUC), which measures the model's ability to distinguish between different classes; Accuracy (Acc), which quantifies the proportion of correct predictions; Sensitivity, which evaluates the model's ability to correctly identify positive instances; Specificity, which measures the model's ability to correctly identify negative instances; Precision, which assesses the proportion of correctly identified positive instances among all instances classified as positive; and the F1 score, which is the harmonic mean of Precision and Sensitivity, providing a single metric that balances. In addition, we have also exploited the G-Mean metric to better illustrate the geometric mean of Sensitivity and Specificity, which is particularly useful for evaluating models in imbalanced datasets.


(4) $$\begin{array}{c} Acc=\frac{TP+TN}{TP+TN+FP+FN}, \end{array}$$

(5) $$\begin{array}{c} Sensitivity=\frac{TP}{TP+FN}, \end{array}$$

(6) $$\begin{array}{c} Specificity=\frac{TN}{TN+FP}. \end{array}$$

(7) $$\begin{array}{c} Precision=\frac{TP}{TP+FP}, \end{array}$$

(8) $$\begin{array}{c} F1\;score=2*\frac{Precision\times Sensitivity}{Precision+Sensitivity}, \end{array}$$

(9) $$\begin{array}{c} G-Mean=\sqrt{Precision\times Sensitivity}, \end{array}$$

where the terms TP (True Positive), TN (True Negative), FP (False Positive), and FN (False Negative) have specific meanings: TP denotes the number of instances where the model correctly predicted the positive class; TN represents number of instances where the model correctly predicted the negative class; FP describes the number of instances where the model incorrectly predicted the positive class when it was actually negative; FN is the number of instances where the model incorrectly predicted the negative class when it was actually positive.

In addition, two more metrics are introduced to provide a deeper understanding of the model's performance: Weighted F1 Score (wF1), which is a variant of the F1 score that takes into account the distribution of the classes. It calculates the F1 score for each class and then averages them, weighted by the number of instances in each class. This is particularly useful when dealing with imbalanced datasets, as it ensures that the performance on the minority class is not overshadowed by the majority class; Weighted Kappa (wKappa), which is a measure of inter-rater agreement that accounts for the agreement occurring by chance. The weighted Kappa extends this concept to the classification task, where it measures the agreement between the predicted labels and the true labels, considering the distribution of the classes. It is particularly valuable in medical image analysis, where it can provide insights into the consistency and reliability of the model's predictions.

### 3.3 Ablation study

To meticulously assess the efficacy of the various components within the proposed model, we executed a series of ablation studies. These studies were designed to measure the performance impact of different inner modules when integrated into the vision transformer architecture. To note that the number of encoders (*E*) denotes the quantity of encoders used in the proposed vision transformer model. And the number of heads (*H*) represents the quantity of attention heads in the proposed encoder module. The results of the ablation studies, which detail the performance of the model with various architectural configurations, are compiled in Table 3 for the APTOS2019 dataset and Table 4 for the Messidor dataset. These tables provide invaluable insights into the contribution of each module and serve as a foundation for understanding the architectural decisions that lead to the most effective model performance in the context of DR grading.

**Table 3:** Outcome of the ablation study on 30% APTOS2019 of the proposed approach with different combinations of *E* and *H*.

**Model**	**Number of encoders (*E*)**	**Number of heads (*H*)**	**AUC (%)**
E_2_H_2	2	2	94.3
E_2_H_4	2	4	94.8
E_4_H_2	4	2	95.1
E_4_H_4	4	4	95.3
E_8_H_4	8	4	95.2
E_8_H_8	8	8	95.8

**Table 4:** Outcome of the ablation study on 30% Messidor of the proposed approach with different combinations of *E* and *H*.

**Model**	**Number of encoders (*E*)**	**Number of heads (*H*)**	**AUC (%)**
E_2_H_2	2	2	94.6
E_2_H_4	2	4	93.9
E_4_H_2	4	2	94.7
E_4_H_4	4	4	95.2
E_8_H_4	8	4	95.8
E_8_H_8	8	8	95.5

Tables 3, 4 demonstrate the performance sensitivity of the proposed model to the number of attention heads and layers. The results reveal that optimizing these parameters can lead to significant improvements in the model's performance across both the APTOS2019 and Messidor datasets. In the context of the DR grading task, the model's proficiency, as gauged by the AUC, Accuracy, wF1, and wKappa metrics, is observed to increase with the incorporation of a greater number of datasets and attention heads. This trend underscores the benefits of an expanded representational capacity, which allows the model to capture more nuanced patterns and relationships within the medical images.

Similarly, for the lesion detection task, there is a discernible enhancement in the AUC, Accuracy, F1 Score, Sensitivity, and Precision when the model is configured with an increased number of layers and attention heads. This suggests that a more complex model structure is better equipped to handle the intricacies of the detection task, leading to more accurate predictions and a more robust classification performance.

The findings from the ablation studies point toward the potential for further performance gains through the introduction of additional training samples and the practice of fine-tuning. By expanding the model's exposure to a broader range of data, and by adjusting the model's parameters to better align with the specific characteristics of the task at hand, it becomes possible to achieve a more refined and effective prediction pipeline. This insight is crucial for guiding future research and development efforts aimed at enhancing the capabilities of the vision transformer for medical image analysis.

### 3.4 Comparison experiments

#### 3.4.1 Lesion detection

Furthermore, to assess the efficacy of our proposed approach in the context of lesion detection, we conducted comparative experiments with state-of-the-art methods on the binary classification task using the entire Messidor dataset. The comparing techniques consist of the followings, Pires et al. (31), CKML Net/LGI (32), CANet (25), Comprehensive CAD (33), DSF-RFcara (34), Expert (33), Multi-task Net (35), MTMR-Net (36), Zoom-in-Net (37), CANet + MultiTask (25), SKD (28), and CNN+Vision Transformer (38). The comparison encompassed a range of performance metrics to ensure a comprehensive evaluation. As indicated by the results, our proposed method exhibited a superior performance across multiple evaluation metrics. Specifically, it outperformed existing state-of-the-art methods in terms of AUC (97.3%), Acc (96.9%), F1 Score (94.7%), Sensitivity (94.1%), and Precision (95.4%), as shown in Table 5. These metrics can reflect the specific models' ability while providing a more subtle understanding of its classification capabilities. To note that we calculated 95% confidence intervals for the performance metrics of our model and the other models. Confidence intervals provide a range within which we can expect the true population parameter to lie with a certain level of confidence, offering a measure of precision for our estimates.

**Table 5:** Comparison between the state-of-the-art techniques and the proposed method on entire Messidor dataset (%).

**Model**	**AUC**	**Acc**	**F1 score**	**Sensitivity**	**Precision**	**G-Mean**
Pires et al. (31)	86.3	-	-	-	-	-
CKML Net/LGI (32)	89.1	89.7	-	-	-	-
CANet (25)	89.5	81.0	-	-	-	-
Comprehensive CAD (33)	91.0	-	-	-	-	-
DSF-RFcara (34)	91.6	-	-	-	-	-
Expert (33)	94.0	-	-	-	-	-
Multi-task Net (35)	94.8	89.9	87.7	85.7	89.7	87.7
MTMR-Net (36)	94.9	90.3	88.3	86.7	90.0	88.3
Zoom-in-Net (37)	95.7	91.1	-	-	-	-
CANet + MultiTask (25)	96.3	92.6	91.3	92.0	90.6	91.3
SKD (28)	96.8	96.9	93.0	93.3	92.7	93.0
CNN+Vision Transformer (38)	97.1	96.5	94.5	93.8	95.2	94.5
Our proposed model	97.3	96.9	94.7	94.1	95.4	94.7

Acc. denotes accuracy.

The performance in AUC indicates that our model has a better ability to rank correctly classified instances higher than incorrectly classified ones. The enhanced F1 Score and Sensitivity suggest that our model is more effective in capturing the presence of lesions without overlooking instances that are actually positive. Similarly, the improved Precision reflects the model's capability to confidently predict positive instances with a lower likelihood of false positives.

To ensure an equitable comparison with contemporary algorithms, we also conducted binary classification experiments on a subset of the Messidor dataset. This subset comprised 500 healthy images (DR grade 0) and 500 images with varying degrees of DR (DR grades 1, 2, and 3). The results of these comparative experiments are summarized in Table 6, where the proposed method demonstrates exceptional performance across several metrics, including Acc and Specificity. Overall, the strong performance in Acc and Specificity positions the proposed method as a formidable contender among state-of-the-art algorithms for the task of binary classification in retinal image analysis.

**Table 6:** Comparison between the state-of-the-art techniques and the proposed method on 50% Messidor for classifying DR and healthy images (%).

**Model**	**AUC**	**Acc**	**Sensitivity**	**Specificity**	**G-Mean**
Expert A (33)	92.2	87.8	-	-	-
Expert B (33)	86.5	76.4	-	-	-
Vo and Verma (39)	87.0	87.1	88.2	85.7	86.9
*S*^2^*MTS*^2^ (40)	86.3	86.7	88.7	84.8	86.7
SRC-MT (41)	84.8	85.8	86.4	85.2	85.8
ACCN (42)	96.0	89.8	93.0	86.7	89.8
Odena et al. (43)	96.7	94.7	95.4	95.1	95.2
CNN+Vision Transformer (38)	97.1	95.3	96.6	94.2	95.4
Our proposed model	96.9	95.6	96.2	95.2	95.7

Acc. denotes accuracy.

#### 3.4.2 DR grading

For DR grading, we also conducted a series of comparison experiments on the APTOS2019 dataset, pitting our approach against a variety of state-of-the-art algorithms. The algorithms selected for comparison include ResNet34 (23), DLI (24), CANET (25), GREEN (26), MIL-VT (27), and CNN+Vision Transformer (38). Notably, the ResNet34 model from (23) comes in two variants: one trained on the ImageNet dataset and another specialized on a dataset of retinal fundus images. Similarly, the GREEN model (26) is available in two distinct architectural forms–GREEN-ResNet50 and GREEN-SE-ResNext50. The results of these comparative experiments, as detailed in Table 7, reveal that the proposed approach outperforms the existing state-of-the-art techniques across a set of evaluation metrics. It is noteworthy that the CNN+Vision Transformer (38) surpasses our approach in terms of AUC (98.3%). However, our framework demonstrates a clear advantage in the critical metrics, including Acc, wF1, and wKappa scores. These results underscore the robustness and effectiveness of our proposed framework in the context of DR grading. By outperforming a diverse set of state-of-the-art algorithms, this work establishes itself as a leading approach for the task of DR grading, offering potential benefits for the early detection and management of this prevalent eye condition.

**Table 7:** Comparison between the state-of-the-art techniques and the proposed method on the entire APTOS2019 dataset (%).

**Model**	**AUC**	**Acc**	**wF1**	**wKappa**
ResNet34 (23)	97.0	85.0	84.7	90.2
DLI (24)	-	82.5	80.3	89.5
CANeT (25)	-	83.2	81.3	90.0
GREEN-ResNet50 (26)	-	84.4	83.6	90.8
GREEN-ResNetNext50 (26)	-	85.7	85.2	91.2
MIL-VT (27)	97.9	85.5	85.3	92.0
CNN+Vision Transformer (38)	98.3	89.1	87.8	91.8
Our proposed method	98.2	89.7	88.1	92.3

Acc represents accuracy, wF1 denotes weighted F1 score, and wKappa denotes Weighted Kappa.

The proposed model achieves an overall Acc of 89.7%, wF1 of 88.1%, and a wKappa of 92.3%. The high values of Acc, wF1, and wKappa indicate that our model is highly effective in grading DR, even in the presence of class imbalance. Class imbalance can often lead to a model that is biased toward the majority class, resulting in poor performance for the minority classes. However, our model's performance suggests that it is capable of accurately grading both common and rare DR grades, which is crucial for early detection and appropriate treatment. The ability of our proposed approach to handle imbalanced classification tasks is a significant advantage, as many real-world applications, including medical image analysis, often deal with such challenges. By providing accurate and reliable predictions across all DR grades, our model demonstrates its potential value in practical applications, contributing to better-informed clinical decisions and improved patient outcomes.

In addition, to demonstrate the generality of the proposed approach, we further conducted comparison experiments between the state-of-the-art deep learning methods and ours on the mini-ImageNet dataset (44), which contains 100 categories of 60,000 color images with 600 images per category. The experimental results are provided in Table 8.

**Table 8:** The comparative results of the state-of-the-art deep learning models and the proposed method on the mini-ImageNet dataset (%).

**Method**	**Precision**	**Sensitivity**	**F1 score**	**AUC**	**G-mean**
MobileNet v3 (45)	83.0	81.9	82.0	99.1	82.4
Desnenet 121 (46)	78.2	77.3	77.3	89.8	77.7
Shufflenet v2_x10 (47)	81.5	81.1	81.0	99.4	81.3
Resnet34 (23)	81.2	80.5	80.6	99.1	80.8
MobileViT (48)	83.4	82.1	82.1	99.4	82.7
Our work	85.2	84.9	84.8	99.5	85.0

These comparative results reveal that the proposed approach is superior over the state-of-the-art methods on mini-ImageNet in terms of Precision, Sensitivity, F1-score, and AUC.

**Figure 6:**
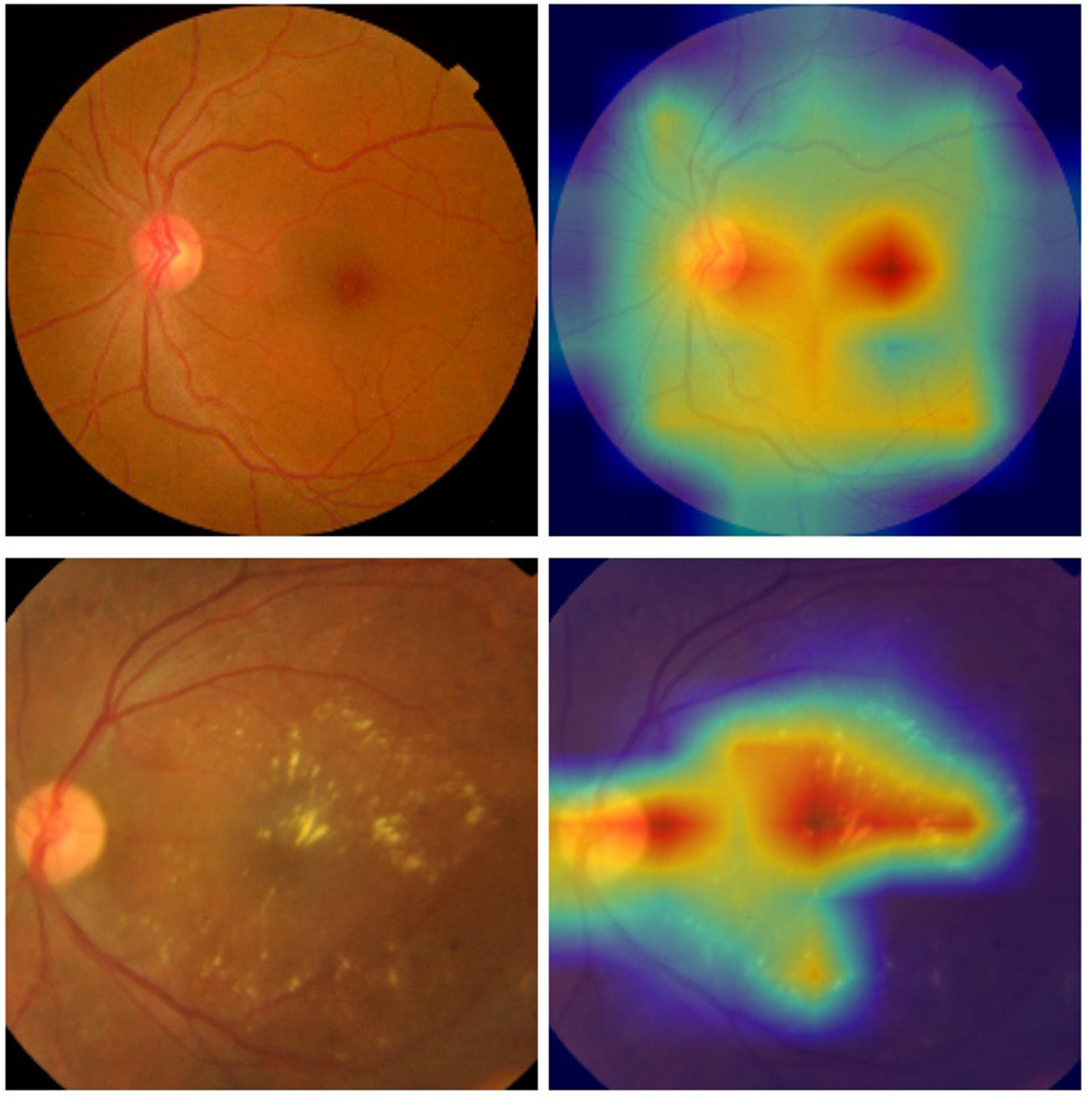
Examples of the retinal images and their corresponding heat maps in the ISIC 2019 dataset using the proposed approach [**(top)** DR 0; **(bottom)** DR 2].

Furthermore, we have leveraged the visual technique Grad-CAM to highlight the regions of the retinal images that the model focuses on when making its predictions, as shown in Figure 6.

#### 3.4.3 Discussion

In this study, we propose that vision transformer focuses predominantly on features associated with the class token, potentially overlooking valuable information extracted from individual image patches. Each patch carries significant relative information that, when utilized, can enhance classification accuracy. The class token's feature representation in the traditional vision transformer would benefit from additional context from the image patches, especially in clinical settings where lesions can be present anywhere in a retinal image. It's important to remember that transformers were initially designed for sequential data processing, and each image patch in the proposed model retains the position information of the whole image. In addition, the proposed integration of softmax and pooling operations into the self-attention module plays a vital role in unveiling the global receptive field while decreasing computational resources. It contributes to implementing the trade-off between expressiveness capability and the quadratic complexity of the attention mechanism. To be more specifically, softmax attention is a fundamental component of the transformer architecture, known for its ability to model global dependencies between elements in the input sequence. It calculates attention weights as a softmax function of the similarity scores between query and key vectors, ensuring that all elements contribute to the output in a weighted manner. Thus, this allows the model to capture complex relationships and dependencies within the data, which is crucial for tasks like image classification where global context is important. On the other hand, pooling is a technique commonly used in CNN models to reduce the spatial dimensions of the representation, thereby reducing the number of parameters and computations required in subsequent layers. In the context of attention mechanisms, pooling is used to downsample the query and key vectors while generating the global and local features of the input. By reducing the dimensionality of the input, pooling helps to decrease the computational complexity of the attention calculations, making the model more efficient without sacrificing the quality of the extracted features. To note that the proxy attention mechanism integrates these two operations by first using pooling to downsample the query and key vectors, creating proxy tokens that capture both global and local features. These proxy tokens are then used in the softmax attention calculation, where the reduced dimensionality leads to lower computational costs during the attention weight computation.

The experimental findings suggest that the proposed vision transformer model, with its unique configuration, is adept at capturing local feature embeddings and identifying global associations. This enables the proposed model to convert retinal images into a detailed feature map, which is crucial for detecting lesions. Furthermore, the streamlined model structure presented in this study is compatible with other vision transformer variants and can be integrated in a modular manner. The proposed model can be integrated into existing electronic health record systems or diagnostic software platforms used in clinical settings. The model can be deployed as a backend service that receives retinal images, processes them, and returns the grading results. These results can then be presented to the healthcare professionals alongside other relevant patient data. Furthermore, in practical applications of the proposed approach in clinical settings, our model offers at least the following advantages over existing systems. Firstly, the proposed approach allows for more accurate detection and grading of diabetic retinopathy lesions, which plays vital role in clinical diagnosis. Secondly, the proposed attention mechanism that integrates both softmax and linear attention, can guarantee the model's ability to capture both global and local features within the retinal images. This attention approach could potentially lead to fewer false negatives and false positives, while improving the overall accuracy and reliability of DR screening. Thirdly, we have focused on optimizing our model for computational efficiency, which is crucial for real-world applications where timely processing of images is essential. Therefore, the proposed model's efficiency allows it to be deployed on various platforms, from high-end servers to edge devices, making it accessible to a wider range of clinical settings, including those with limited resources.

However, there are still several limitations to this study. Firstly, the quantity of data samples is limited, which could impact the performance of deep learning models, as the number of images is directly related to model outcomes. Secondly, the range of evaluation metrics employed is limited, and expanding the set of metrics used could provide a more comprehensive assessment. In addition, the proposed model has been optimized for efficiency, with an average processing time of 420 milliseconds per image during our experiments. This performance indicates that the model is capable of providing near real-time feedback, which is essential for clinical decision-making processes. However, it is notable that the current version of the proposed model may not meet the real-time performance requirements for all clinical settings, especially those with limited resources. Therefore, we are committed to further optimizing this model to ensure it can be deployed in a wide range of environments, from well-equipped hospitals to remote clinics with basic infrastructure.

## 4 Conclusion

To tackle the challenge of classifying retinal images, we have integrated a newly proposed attention mechanism into the conventional vision transformer model. This research investigates the potential of integrating linear and softmax attention modules for the task of identifying retinal lesions. While CNNs have made significant strides in image classification by revealing intrinsic image properties, they tend to concentrate on local features. In contrast, vision transformers offer a broader perspective by utilizing feature embeddings from CNNs to grasp the global context. After pre-training on a large-scale natural image dataset and fine-tuning with retinal image datasets, this hybrid model has demonstrated its proficiency in detecting retinal lesions and has outperformed leading CNNs and vision transformers.

For future endeavors, we plan to experiment with integrating different backbone networks as feature extractors and exploring various classification algorithms. Given the promising results for retinal image analysis, we aim to apply the proposed architecture to classify a broader range of images.

## Data Availability

Publicly available datasets were analyzed in this study. This data can be found here: https://riadd.grand-challenge.org/Download/ RFMiD 2.0; https://www.kaggle.com/datasets/mariaherrerot/aptos2019 APTOS2019; https://www.adcis.net/en/third-party/messidor/ Messidor.

## Footnotes

^1^ https://www.image-net.org/challenges/LSVRC/index.php
